# Efficacy of LSVT LOUD^®^ on Phonatory Control and Voice Quality in Patients with Primary Progressive Apraxia of Speech: Case Studies

**DOI:** 10.3390/brainsci14050417

**Published:** 2024-04-24

**Authors:** Yee Nam Candice Choi, Vincent Martel-Sauvageau, Myriam Breton, Monica Lavoie, Robert Laforce, Liziane Bouvier

**Affiliations:** 1 School of Communication Sciences and Disorders, McGill University, Montréal, QC H3A 0G4, Canada; yee.choi@mail.mcgill.ca; 2 Faculté de Médecine, Université Laval, Québec, QC G1V 0A6, Canada; vincent.martel-sauvageau@fmed.ulaval.ca (V.M.-S.); robert.laforce@fmed.ulaval.ca (R.L.J.); 3 CIRRIS—Centre Interdisciplinaire de Recherche en Réadaptation et Intégration Sociale, Québec, QC G1M 2S8, Canada; myriam.breton.2@ulaval.ca; 4 CHU de Québec-Université Laval, Québec, QC G1V 0A6, Canada; 5 Clinique Interdisciplinaire de Mémoire, Hôpital de l’Enfant-Jésus, Québec, QC G1J 1Z4, Canada; monica.lavoie.1@ulaval.ca; 6 Chaire de Recherche sur les Aphasies Primaires Progressives—Fondation de la Famille Lemaire, Université Laval, Québec, QC G1V 0A6, Canada; 7 CRIR—Centre for Interdisciplinary Research in Rehabilitation, Montréal, QC H3S 1M9, Canada

**Keywords:** PPAOS, progressive apraxia of speech, LSVT, hypophonia, voice, treatment

## Abstract

Primary progressive apraxia of speech (PPAOS) is a neurodegenerative syndrome characterized by the progressive and initially isolated or predominant onset of difficulties in the planning/programming of movements necessary for speech production and can be accompanied by dysarthria. To date, no study has used an evidence-based treatment to address phonation control in patients with PPAOS. The aim of this study was to evaluate the feasibility and efficacy of LSVT LOUD^®^ as a treatment for phonatory control in speakers with PPAOS. Three speakers with PPAOS received LSVT LOUD^®^ therapy, and changes in phonatory control, voice quality and prosody were measured immediately, and one, four and eight weeks after the end of the treatment. Overall, the results suggest that the treatment is feasible and could improve voice quality, intensity, and control in some patients with PPAOS. The generalization of the results is also discussed.

## 1. Introduction

Primary progressive apraxia of speech (PPAOS) is a neurodegenerative syndrome characterized by the progressive and initially isolated or predominant onset of difficulties in the planning/programming movements necessary for speech production. Since its first appearance in the literature in 2006 [1], PPAOS has been progressively recognized as a distinct syndrome from non-fluent primary progressive aphasia. A growing number of studies have focused on the motor speech and neuroanatomical correlates of PPAOS. As expected in apraxia of speech, articulatory and prosodic impairments have been described in PPAOS [2,3,4,5,6,7,8,9], with prosody mostly affected in terms of the timing of speech (speaking rate, reduced number of syllables per breath groups, syllabification, and abnormal rhythm). Reduced variations in pitch during sentence production in patients with predominant apraxia of speech and concomitant aphasia have also been reported [2,10,11]. Finally, co-occurring dysarthria would be present in up to 30% of patients with PPAOS, with the most frequent types being spastic, hypokinetic, or mixed spastic–hypokinetic [12,13,14]. Clinical experience also suggests that some patients present with dysphonia or difficulties in phonatory control.

While most speakers with PPAOS present with both phonetic and prosodic abnormalities, there is some heterogeneity in their motor speech profiles. This led to the distinction of three distinct PPAOS subtypes, which are categorized based on the relative predominance of speech patterns [5,12,13]. The phonetic subtype is characterized by a predominance of articulatory distortions, distorted sound substitutions or additions and articulatory groping and attempts at self-correction of phonetic-level errors. The prosodic subtype is characterized by a predominance of slow rate and segmentations between words or between syllables in multisyllabic words. The mixed subtype is characterized by the lack of predominance between phonetic and prosodic abnormalities and is mostly assigned in very mild or very severe cases. Different disease trajectories would be related to the different subtypes. The phonetic subtype is associated with faster rates of decline in motor speech and aphasia, and evolution into frontotemporal dementia. The prosodic subtype is associated with faster rates of decline in non-speech motor function, onset of Parkinsonian features, and evolution into Parkinsonian syndromes [4,7,13,15,16].

The growing interest into PPAOS has led to a better understanding of this syndrome. However, very few studies have examined interventions in speakers with progressive apraxia of speech (PAOS) [17]. Pattee, Von Berg, and Ghezzi [18] aimed to evaluate alternative modes of communication, namely text-to-speech augmentative and alternative communication and American Sign Language, to a patient presenting with primary progressive aphasia (PPA) and apraxia of speech (AOS) whose speech was characterized as minimally intelligible. Following treatment, the patient demonstrated significant improvements in communicative effectiveness across all measures. Rogalski et al. [19] investigated the feasibility of using tele-practice as a method of providing treatment to individuals with a clinical diagnosis of dementia presenting with prominent aphasia symptoms. The internet-based treatment garnered overwhelmingly positive feedback, resulting in significant improvements in cognitive-communication domains and communication confidence. In a single-subject study with a patient with AOS and aphasia, Henry et al. [20] used structured oral reading as a strategy to improve the production of multisyllabic words. Gains in multisyllabic word production and increased self-corrections of speech errors were reported for both trained and untrained texts. According to Western Aphasia Battery (WAB) scores gathered at one-year follow-up, AOS ratings worsened, and a decrease in fluency rating was observed (demonstrating the progressive nature of PAOS). Despite this, participants indicated improved confidence in communication, as well as improved performance and comfort in reading aloud and speaking, according to post-treatment surveys. The effectiveness of structured oral readings as a tool for improving speech production was further investigated by Machado et al. [21] Following treatment, participants presenting with non-fluent PPA (nfPPA) demonstrated significant improvement in trained and untrained texts as well as a significant reduction in articulatory errors, concluding that structured oral reading is an efficient and effective method of addressing multisyllabic word production in AOS associated with nfPPA. Henry et al. [22] examined the effectiveness of video-implemented script training for aphasia (VISTA) for individuals diagnosed with nfPPA with features of AOS, specifically targeting articulatory and grammatical aspects of script production. According to post-treatment evaluations, participants demonstrated significant improvement in overall percent intelligibility and correctness, as well as a significant reduction in grammatical errors in trained scripts. Gains for trained scripts were maintained for up to one year, while measures for untrained reading scripts remained stable during follow-up sessions. In a single-case study with a patient with nfPPA with AOS presenting with difficulty initiating speech, Beber et al. [23] aimed to examine the importance of rate and rhythm strategies in alleviating AOS in nfPPA. For medical reasons, no objective post-treatment assessment was conducted; outcomes were gathered qualitatively through patient and partner observations and reports. However, qualitative reports and information from the participant and their partner indicated that rate and rhythm strategies allowed them to initiate speech in many situations and produce single words and short sentences.

The results of these studies are difficult to generalize due to the variance in clinical diagnoses (concomitant or predominant aphasia, or concomitant dysarthria), the low level of evidence in the diagnosis [17], and focus of the treatment (articulation, rate and rhythm, language, non-oral communication). Moreover, the strength of the evidence is limited because the level of evidence for the treatment effectiveness was low [17] and because the treatment was not based on the motor learning principles recommended in the treatment of apraxia of speech [24,25]. Although promising, these treatments only address a portion of the known deficits in PPAOS, namely articulation disorders and speaking rate and rhythm. No study has targeted the phonatory-prosodic difficulties encountered in these patients (e.g., decreased intonation modulation, difficulties in managing breathing groups, altered vocal quality).

Considering the degenerative nature of PPAOS and the limited number of patients available, it is indicated to use a recognized treatment for speech disorders in a neurodegenerative context that is proven to be effective and whose effects last over time. The Lee Silverman Voice Treatment (LSVT LOUD^®^) was initially developed to treat hypophonia (low voice intensity) in patients with Parkinson’s disease [26]. Based on the fundamental principles of motor learning, this therapy uses a progressive feedback approach based on performance and level of progression to elicit a patient’s production, reflecting a loud voice through structured and repetitive tasks. The effort given to increase the intensity of the voice would generate a general motor activation that would also activate the rest of the speech motor centers. Indeed, by speaking louder, articulatory gestures would be larger and more supported, production would be more stable, intonation modulation would be accentuated, and breathing and speech coordination would be facilitated [27,28,29].

Our interest in investigating the effect of LSVT LOUD^®^ on phonatory control in patients with PPAOS is based on evidence of its beneficial effects on voice modulation, phonatory control, and speech systems coordination—including respiration and phonation [30,31] and its efficacy in patients with other conditions relevant to PPAOS. The efficacy of LSVT LOUD^®^ has been demonstrated not only for patients with Parkinson’s disease or Parkinsonian syndromes but also for ataxic dysarthria [32], post-stroke dysarthria [33], and multiple sclerosis [34], conditions all sharing clinical or neuropathological features with PPAOS, including articulatory and prosodic impairments (imprecise articulation, slow rate of speech, prolonged syllables) and the involvement of cortical and subcortical connections and structures (e.g., superior cerebellar peduncle, cortical-projection fibers [35]).

Because of its well-documented effect on multiple speech components (phonation, articulation, prosody, respiration), its motor learning theoretical foundation, and its efficacy in other neurodegenerative disorders sharing important characteristics with PPAOS, evaluating the efficacy of LSVT LOUD^®^ on phonatory control in patients with PPAOS is a first step towards the establishment of targeted and effective therapies for the rehabilitation of motor speech disorders in patients with progressive apraxia of speech.

## 2. Materials and Methods

### 2.1. Participants

A total of four participants were recruited. For unrelated medical reasons, one of them withdrew before starting the treatment sessions and was therefore excluded from this study. All had received a neurological diagnosis of PPAOS following neurological and speech-language pathology evaluation, based on the criteria described by Duffy and colleagues [12]. AOS was determined based on the presence of commonly recognized symptoms, including but not limited to labored speech production, inconsistent speech sound errors, distortions, disrupted prosody [12,24]. Furthermore, two of the authors who also are speech-language pathologists (L.B., V.M.-S.) independently reviewed the audio recordings from the first baseline session. They agreed about the presence of AOS based on [12,24] and the Apraxia of Speech Rating Scale (ASRS) 3.5 [36]. Detailed results of the ASRS 3.5 for each participant are presented in the case descriptions below (Section 2.1.1, Section 2.1.2 and Section 2.1.3). To be included in this study, the participants needed to present with difficulties in phonation control or quality, confirmed by a speech-language pathologist. The independent review of the audio recordings by L.B. and V.M.-S. also confirmed alterations in phonation control or voice quality. Results of the G.R.B.A.S (0 = normal, 1 = mild, 2 = moderate, and 3 = severe [37]) are presented in the case descriptions below (Section 2.1.1, Section 2.1.2 and Section 2.1.3). Due to the nature of the treatment, unequivocal spastic dysarthria was an exclusion criterion.

All participants were native Quebec French speakers and had normal or corrected vision and hearing. They had a smoking history, but all had successfully quit 20 to 40 years prior. No participant reported any health or medication changes over the course of the treatment. None of our patients received other speech therapy over the course of this study.

#### 2.1.1. Participant LSVT01

Participant LSVT01 is a 73-year-old female who began exhibiting motor speech symptoms two to three years prior to the start of treatment, characterized by changes in vocal quality and speech intelligibility. She reported difficulty articulating long or complex words—with omissions of syllables in multisyllabic words—and linking speech sounds, reduced articulation speed, and occasional difficulties with speech fluidity, including initiation difficulties, repetitions and word searching difficulties. She also presented monotonous intonation, ‘like a robot’, as well as fatigue and vocal hoarseness that increased over the course of the day. No prior medical problems were reported.

**Figure 1:**
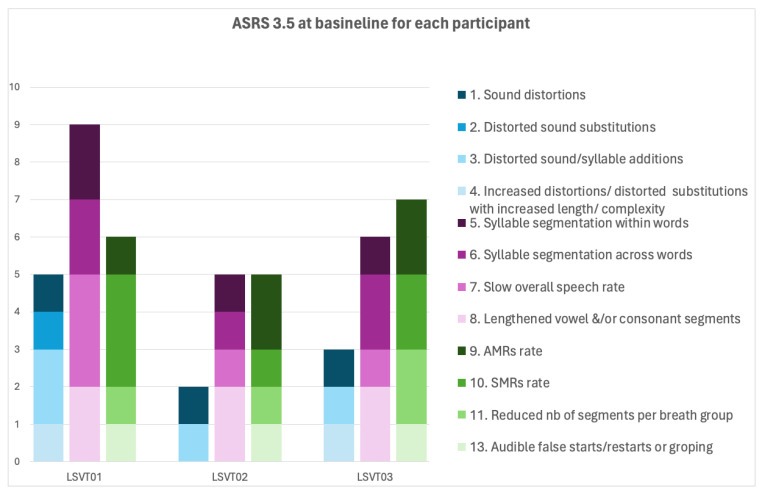
Results of the ASRS 3.5 for all participants at the first baseline visit. Item 12 of the scale (silent articulatory false starts/restarts or groping) was not rated since no video recording was available.

**Figure 2:**
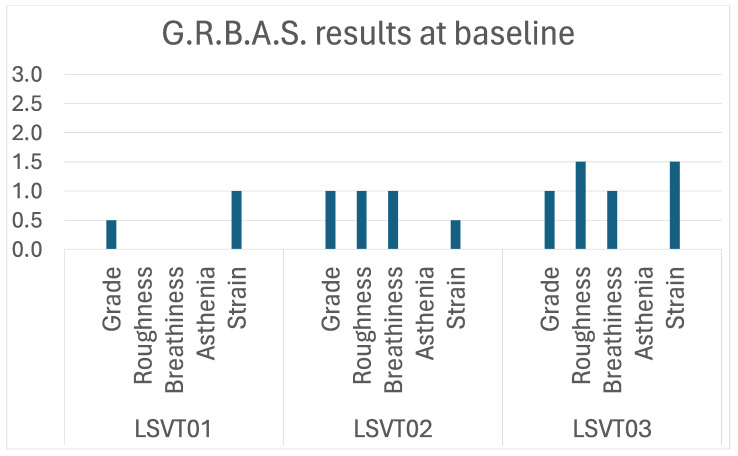
Results of the G.R.B.A.S. for all participants at the first baseline visit.

Figure 1 and Figure 2 present the results of the motor speech assessment at baseline. The ASRS 3.5 revealed a greater score in prosodic (9) than in articulatory features (5), and a total score of 20 (cut-off for AOS in English speakers: 10 [36]). More specifically, she had occasional sound distortions and distorted additions and frequent schwa intrusions. She had frequent within- and across-word syllable segmentation and sound lengthening and significantly reduced speaking rate. She had mildly reduced breath groups (6–7 syllables). She had greater difficulties with SMRs than AMRs. She also had infrequent false starts and repetitions. The patient had difficulties with the control of intensity and pitch. Her voice quality was mildly reduced, with a score GRBAS [37] score of G_0.5_R_0_B_0_A_0_S_1_. Despite occasional weakness in her voice, she presented with increased intensity when speaking with increasing effort, for example, when trying to produce DDKs.

#### 2.1.2. Participant LSVT02

Participant LSVT02 is a 75-year-old male who began exhibiting motor speech symptoms three years prior to the start of treatment, characterized by articulation difficulties and changes in voice quality. He reported articulation difficulties, specifically with the speech sound /ʁ/ and the production of tri- or quadrisyllabic words and unfamiliar words, slowness of speech, difficulty initiating words, and increased fatigue at the end of the day. He also indicated that his voice had become ‘scratchy’ and weaker. He had been experiencing bilateral hearing difficulties for approximately 10 years. He also reported a possible transient cerebral ischemia a few years prior, without any motor speech sequela.

Figure 1 presents the results of the motor speech assessment at baseline. The ASRS 3.5 revealed a greater score in prosodic (5) than in articulatory features (2) and a total score above the cut-off (12). The patient presented with mild articulatory impairments and more prominent prosodic impairments, characterized predominantly by lengthened sounds and syllable segmentation across words. Voice quality was mildly reduced, with a GRBAS [37] score of G_1_R_1_B_1_A_0_S_0.5_.

#### 2.1.3. LSVT03

Participant LSVT03 is an 82-year-old male who noticed progressive changes in his speech two years prior to the start of treatment. He reported difficulties «getting the words out», increasing hesitations, and sometimes getting out of breath when speaking. He also developed mild hoarseness and faintness of voice as well as occasional diplophonia. No other health issues were reported.

Figure 1 presents the results of the motor speech assessment at baseline. The ASRS 3.5 revealed a greater score in prosodic (6) than in articulatory features (3) and a total score (16) above the cut-off. The patient presented with mild articulatory impairments and more prominent prosodic impairments characterized predominantly by lengthened sounds and syllable segmentation across words. Voice quality was mildly reduced, with a GRBAS [37] score of G_1_R_1.5_B_1_A_0_S_1.5_.

### 2.2. Treatment Protocol

All participants received treatment in accordance with LSVT LOUD^®^ procedures [26]. Each participant was given 60 min treatment sessions 4 days per week for four weeks, for a total of 16 sessions. All treatment sessions were performed remotely through Zoom (Version 5.5.2 and above) by an independent LSVT LOUD^®^ certified speech-language pathologist. Participants connected to the sessions from home, and the SLP connected to the sessions from a remote location using their local Internet connection. Participants were asked to be in a quiet room to reduce background noise and were provided with an external microphone (SF-666 Cardioid Condenser Microphone, ZaxSound, Shenzhen, China). To minimize audio enhancements applied by Zoom, “Suppress background noise” was set to “Low,” “High fidelity music mode” was enabled, and the “Original sound” feature was used during tasks involving sustained phonation. Participants were asked to use the same equipment and setup for all sessions in order to reduce potential within-participant variability between sessions.

### 2.3. Evaluation of Efficacy

To evaluate the efficacy of treatment, separate visits were conducted by a trained speech-language pathologist who was blinded to the specific aims of this study and did not administer the therapy. Baseline data were collected on three consecutive days prior to the start of treatment (Pre-1, Pre-2, Pre-3). Immediately post-treatment data were collected on three consecutive days following the end of treatment (Post-1, Post-2, Post-3). Follow-up data were collected on three blocks of three consecutive days at 1 week (FU1-1, FU1-2, FU1-3), four weeks (FU2-1; FU2-2, FU2-3) and eight weeks (FU3-1; FU3-2, FU3-3) after the end of the treatment. LSVT01 and LSVT02 completed all evaluation visits in person and were recorded using a Tascam DR-40 audio recorder (Tascam, Shenzhen, China) and a Shure SM-10A head-mounted microphone (Shure, Juárez, Mexico; fixed 4 cm mouth-to-microphone distance). LSVT03 was evaluated through Zoom, using the same technological setup as during the treatment sessions (see above).

The evaluation protocol included trained tasks and items that were targeted during treatment (sustained vowel phonation, pitch glides, personalized functional sentences, and conversation) and untrained tasks and items that were not targeted during treatment (standard functional sentences, standard passage reading, picture description, and diadochokinesias [DDK]). Untrained tasks aimed to assess the generalization and integration of gains outside of trained items. Treatment adherence and perceptions of the treatment outcomes were collected from the patient per- and post-treatment.

The primary outcome variables consisted of selected acoustic measures of vocal function during trained tasks: mean intensity during sustained vowel, personalized sentences and conversation; maximum and maximum F0 and F0 range during pitch glides; smoothed cepstral peak prominence (CPPS), jitter, shimmer, and harmonic-to-noise ratio (HNR) during sustained vowel. Secondary outcome variables consisted of selected measures of vocal function during untrained tasks as well as measures of prosody and respiratory-phonatory coordination: mean intensity during standardized sentences and picture description; acoustic voice quality index (AVQI) during passage reading, coefficient of variation (CV) of F0 and intensity during connected speech, and speech and pause measures during connected speech (speaking rate, length of articulatory groups, mean speech duration, percentage of pause).

### 2.4. Acoustic Analyses

Changes in speech abilities across various speech systems were captured using acoustic measures. See Table 1 for details on targeted abilities, acoustic measures, and associated tasks. All acoustic analyses were conducted using Praat software v6.3.08 running on Mac OS by trained research assistants blinded to the timing of the sessions and the aims of the treatment [38]. Ten percent of all analyses were analyzed for interrater analysis using intraclass correlation analyses (two-way mixed-effects, single or average measures, as relevant). All results ranged from good to excellent (0.846 to 1.00) [39].

#### 2.4.1. Phonation

Average loudness. Mean intensity (in dB SPL) was measured in five tasks: sustained phonation, functional and standardized sentences, picture description, and conversation. In the sustained phonation task, the mean intensity (in dB SPL) was measured from the onset to the offset of phonation—determined by the first and last visible glottal pulse on the oscillogram—using the Praat command “Get intensity”. In the standard and functional sentences, the mean intensity was measured by averaging the peak intensity of each vowel, which was identified through an automatic script [40], manually corrected, and then averaged across the whole task. In the picture description and conversation tasks, mean loudness was measured by averaging the intensity peak—of the vowel—of the last syllable of each verb. As verbs generally take stressed positions in sentences, this procedure aimed to standardize the elements of loudness across productions with varying content [28].

Control of pitch. The upward pitch glide task consisted of gliding the pitch during the sustained production of the vowel/a/, from the participant’s neutral/habitual pitch to the highest pitch possible and holding this pitch for 2 s. The downward pitch glide task consisted of gliding the pitch from neutral/habitual pitch to the lowest pitch. Maximum and minimum pitch were measured as the maximum or minimum stable pitch (held at least 500 ms) achieved in the second half of the vowel (measured in Hz). If no stable pitch was found at the end of the vowel, the mean value around the highest value was calculated (500 ms), excluding outlying data due to voice breaks or octave jumps. The downward and upward pitch ranges were calculated as the difference in semitones between the neutral pitch and the minimum or maximum pitch, respectively, during the stable pitch portion of the second half of the vowel.

Voice quality. The AVQI is a multiparametric approach that includes six acoustic parameters to qualitatively assess and compute overall voice quality using an automated Praat script [41,42]. The parameters include 6 measures that are time-related (HNR, shimmer [%] and shimmer [local] dB), frequency-related (general slope of long-term average spectrum, tilt of regression line through long-term average spectrum) or quefrency-related (CPPS). The AVQI is adapted for both sustained phonation and connected speech tasks. The output is a score between 0 (normal voice) and 10 (severely dysphonic), where the cut-off threshold is 2.33 for French speakers [41]. The analyzed speech sampled three seconds of sustained phonation and three seconds of connected speech from the passage reading task [43]. Individual parameters of voice quality frequently used in clinical settings were also extracted on the sustained vowel only: CPPS, jitter, shimmer, and HNR.

#### 2.4.2. Prosody

Speech timing. Speech timing measures were obtained from the passage reading task. Speaking rate, in syllables per second, was calculated by dividing the total number of syllables produced by the total duration of the task—from beginning to end of speech. The percentage of pauses was calculated by dividing the sum of the duration of all pauses of at least 200 ms during the task by the total duration of the task. Mean speech duration was obtained by averaging the duration of all speech segments (speech segments delimitated by pauses of at least 200 ms).

Variations in pitch and loudness during speech. The coefficient of variation (standard deviation/mean) of pitch in semitones and intensity in decibels (dB) were measured over the standardized and personalized sentences, the passage reading task and the conversation, using only the voiced speech segments (concatenated). Pitch tracking was manually corrected.

#### 2.4.3. Articulation

Maximum articulation rate. DDK rate for the syllables /pa/, /ta/ and /ka/, and the sequence /pataka/ were calculated in syllables per second, i.e., the number of syllables produced during the first five seconds of the task, divided by 5 s. The final value for each stimulus represents the best of three trials.

### 2.5. Statistical Analysis

Tau-U was used to analyze our data as it takes on a non-parametric approach that is appropriate for small data sets commonly seen in single-subject research [44]. Tau-U is a method for qualitatively analyzing single-case experimental data that enhances visual analysis and incorporates significance testing. Tau-U assesses non-overlap between baseline and treatment phrases, as well as trends from within the treatment phase [45]. In addition, Tau-U has the advantage of estimating and correcting for a baseline trend in its calculation, which is useful to account for variable performance and the potential for unwarranted baseline trends, which are highly characteristic of populations like those with aphasia and PPAOS [44].

### 2.6. Missing Data

Due to an illness unrelated to this study, LSVT01 completed only 2 FU3 visits. Due to technical issues, the recording for LSVT02 conversation task at visit FU1-3 is unavailable. Personalized sentences were not recorded during the pre-treatment phase for LSVT02. LSVT03 produced only 2 usable sustained vowel repetitions at visit FU2-1.

## 3. Results

Tau-U results for each participant for each speech component are presented in the Supplementary Materials (Tables S1–S3). For clarity and conciseness, only the significant results are presented in the next sections. Results are presented by participants below, and are summarized in Table 2, Table 3 and Table 4.

### 3.1. LSVT01

#### 3.1.1. Primary Outcomes

**Figure 3:**
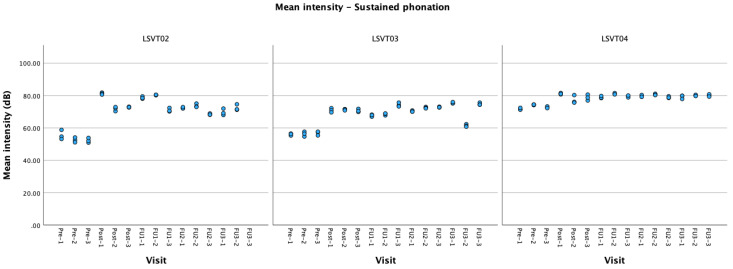
Mean intensity values (in dB) during sustained phonation for all participants at each visit.

Intensity. There was a significant increase in voice intensity during all trained tasks (sustained phonation, personalized sentences and conversation) at post-treatment. Improvement in sustained phonation (see Figure 3) and personalized sentences remained significant over all follow-up visits; conversation task remained significant up to four weeks, and was near significance at the eight weeks follow-up.

Phonation control. There was a significant improvement in minimum pitch and pitch range that remained significant at the eight-week follow-up.

**Figure 4:**
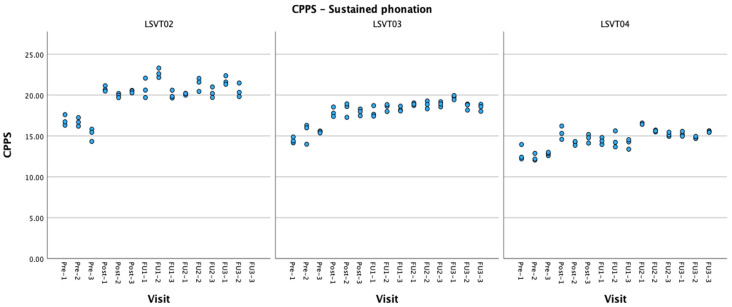
CPPS values during sustained phonation for all participants at each visit.

Voice quality. There was a significant increase in CPPS values at post-treatment and all follow-up sessions (see Figure 4). The patient was close to or above the cut-off at pre-treatment sessions (range: 14.33–17.21; cut-off CPPS for vowels: 14.45, Murton et al., 2020 [46]), but their voice values still improved clearly above the cut-off after treatment and were maintained during follow-up (range: LSVT01: 19.65–23.30). HNR, shimmer, and jitter values all improved and remained significant over all follow-ups.

#### 3.1.2. Secondary Outcomes

Intensity. Voice intensity during the standardized sentences and picture description was increased immediately post-treatment. It remained significant at all follow-ups for the sentences, significant up to four weeks for picture description, and near significant at the eight-week follow-up.

Voice quality. The participant had a variable AVQI across pre-treatment sessions (range: 1.96–3.80) that crossed the maximum cut-off (2.33 [41]), but the values stabilized after treatment and remained below 1.20 at post-treatment sessions and below 1.54 at follow-up sessions—well below the cut-off.

Prosody. There was a trend towards shorter mean pause duration for LSVT01 that did not reach significance. There was a significant increase in loudness variations in standardized sentences and picture description, which was no longer significant at the 4 weeks (sentences) and 8 weeks (picture description) follow-ups. There was a significant increase in pitch variations during standardized sentences at all visits.

Articulation. There was no significant change in maximum articulation rate.

### 3.2. LSVT02

#### 3.2.1. Primary Outcomes

Voice intensity. Voice intensity in sustained vowels was significantly increased at all sessions (see Figure 3). Personalized sentence recordings were not available for baseline.

Phonation Control. There was a significant increase in the pitch range during pitch glides from baseline to post-treatment and follow-ups.

Voice quality. All voice quality measures (CPPS, jitter, shimmer, HNR) were significantly improved at all sessions (see Figure 4). CPPS values were close to or above the cut-off at pre-treatment sessions (range: 14.15–16.29) but their voice quality still improved after treatment and was maintained during follow-up (range: LSVT03: 17.27–19.94).

#### 3.2.2. Secondary Outcomes

Voice intensity. Voice intensity in standardized sentences was significantly increased at all sessions.

Voice quality. AVQI was significantly improved at all sessions.

Prosody. No prosody measure was significant.

### 3.3. LSVT03

#### 3.3.1. Primary Outcomes

Voice intensity. There was an increase in voice intensity during sustained phonation that was maintained at all follow-ups (see Figure 3). There was an increase in voice intensity for personalized sentences post-treatment that was not maintained at follow-up.

Phonation Control. There was an improvement in minimum and maximum pitch, and pitch range post-treatment and at all follow-ups.

Voice quality. Jitter, shimmer and HNR were improved at post-treatment and remained significant at most follow-up visits.

#### 3.3.2. Secondary Outcomes

Voice quality. AVQI during connected speech was improved at all sessions.

Prosody. The coefficient of variation of intensity was increased post-treatment in personalized sentences post-treatment until the 4-month follow-up inclusively. The coefficient of variation of intensity was significantly increased at post-treatment only. Significant mean speech duration increase, and percentage of pause decrease were found at all sessions.

### 3.4. Participant Impressions on Treatment Outcomes

Participant LSVT01 noted that others, including neighbours and relatives, provided positive feedback on speech intelligibility, articulation, and clearness. Furthermore, she expressed satisfaction with speech changes and intends to attend follow-up sessions to maintain the skills obtained. Participant LSVT02 noted that others commented on improved speech intelligibility and clearness. Participant LSVT03 expressed satisfaction with treatment results and noted increased comfortability in public speaking, as well as positive feedback on improved speech intelligibility and loudness from friends.

## 4. Discussion

The aim of this study was to evaluate the feasibility and efficacy of LSVT LOUD^®^ as a treatment for phonatory control and voice quality in speakers with PPAOS. Although not a primary symptom of PPAOS and is not present in all speakers with PPAOS, reduced variations in pitch have been reported in adults with AOS before by our team and others [10,47,48,49]. This case study presents three speakers with PPAOS who received LSVT LOUD^®^ therapy, and for whom changes in phonatory control, voice quality and prosody were measured immediately, 1 week, 1 month, and 2 months after the end of the treatment. All received therapy remotely from a certified LSVT LOUD^®^ clinician. The efficacy of remote and virtual LSVT LOUD^®^ has also been demonstrated in previous studies [50,51,52], confirming the non-inferiority of remote LSVT LOUD^®^ in improving variables such as vocal loudness, sustained vowel phonation and duration, compared to in-person methodologies [50].

Following our expectations, all participants had a significant improvement in the primary outcomes after the treatment. Maintenance of the gains over time was variable. All participants had increased voice intensity during sustained phonation after the treatment and during all follow-ups. The two participants who had personalized (functional) sentences at baseline had increased voice intensity during the task, with variable durability of the gain (only post-treatment and 8 weeks). There was an increase in voice intensity for two participants during the production of the general untrained sentences, but this gain was variable during follow-ups. It is important to note that mild hypophonia was a complaint only for LSVT01, who demonstrated an increased vocal intensity during conversation and picture description tasks. Mild hypophonia was also present for LSVT03 but was not a main complaint and was not reported as having an important functional impact on their communication. The limited generalization of voice intensity gains in the other two patients is somewhat expected as a general increase in voice intensity was not a main concern before the start of the treatment for them.

In the secondary outcomes, generalization to more ecological tasks was limited. Two participants showed improvement in intensity variations during personalized sentences and one during standardized sentences. One participant showed improvement in pitch variations during personalized sentences. There was no significant change in intensity or pitch variations after the treatment for any participant during picture description and conversation. The limited generalization to the more ecological tasks for intensity control and modulations could reflect the important effect of task complexity on the vocal effort that was also reported during treatment for two of the participants. This type of interaction has been reported in patients with Parkinson’s disease during LSVT treatment [28]. Moreover, neurocognitive symptoms, including executive dysfunction [4,7,8,53,54], have been reported in speakers with PPAOS. It is possible that the type of task used here for training the pitch and intensity modulation abilities or for measuring carry-over could be improved. In treatment stimuli, it could be important to control variables that are known to influence speech production in AOS. For example, manipulating the phonetic complexity and length of sentences could help adjust the difficulty to focus more on pitch modulations without overcharging the system with phonetically complex sentences. Moreover, including different types of sentences (interrogative, exclamative, imperative, and neutral) could improve outcomes by exposing the patient more to these types of sentences and guided practice with the clinician. The use of tasks including both linguistic and emotional prosody features could also facilitate the transfer of modulatory abilities acquired in pitch glides to more ecological discourse and have a greater impact on daily life communication. In outcome measures, standardized sentences included two questions. Having a greater number of interrogative sentences would allow more specific assessment of linguistic prosody abilities. Moreover, combining perceptual and acoustic measures would provide a more functional representation of the patient’s abilities.

The results in the ability to modulate the pitch were variable across participants. One participant (LSVT02) had increased maximum F0 during the vowel glide that was maintained over time but no improvement in minimum F0. One participant (LSVT01) had the opposite pattern, with lasting improvement in minimum F0 but no improvement in maximum F0. One participant (LSVT03) demonstrated lasting improvement in both maximum and minimum F0. Results for the range achieved during the glides were inconsistent. This discrepancy between an increase in F0 and no increase in range is explained by a tendency for the participants to start at a higher or lower F0 to realize the upwards and downward glides, respectively. A qualitative assessment also suggests that there was an important improvement in voice quality during the higher frequencies for LSVT02 and an inconsistent improvement for LSVT03, which suggests a better control of phonation. An important limit in the participants’ performance was the reversal of pitch direction. When instructed to do a downward glide, participants would sometimes glide upwards instead, and vice versa. It was sometimes possible for them to achieve the correct direction afterwards with visual cueing (downward hand motion), but sometimes there was persistence of the upward glide on the downward glide. This reversal of direction during voluntary phonation further supports our hypothesis of phonatory apraxia in some patients with PPAOS [10]. Similarly, previous studies have reported difficulties with the coordination of voicing and problems with initiation and termination of voice action [55,56].

All patients improved in at least three measures of voice quality: jitter and shimmer during sustained phonation and AVQI during connected speech. Moreover, at least one measure remained significant over a 2-month follow-up period. Positive effects of LSVT treatment on voice quality have been reported previously [26,32,57,58]. The improvements have been hypothesized to result from better phonatory control or increased awareness of voice quality. This could be the case also in patients with PPAOS. For example, the improvements in objective voice quality measures for participant LSVT03 are in concordance with the perceptual observations reported by the treating SLP: decrease in initiation difficulties on phonation onset, reduced vocal forcing and tension, and increase in self-corrections, as well as improvement in vocal projection. Vocal forcing and tension might arise from spastic dysarthria, which has been reported in PPAOS [10,12] or phonatory apraxia [10]. Increased control of voluntary phonation could result in the observed improvements in voice quality and decreased occurrence of glottal attacks at the initiation of phonation. Similar to what was found for the voice intensity outcomes, the patient who presented the most generalized and lasting improvement was also the one who presented with the lowest voice quality on the GRBAS.

**Figure 5:**
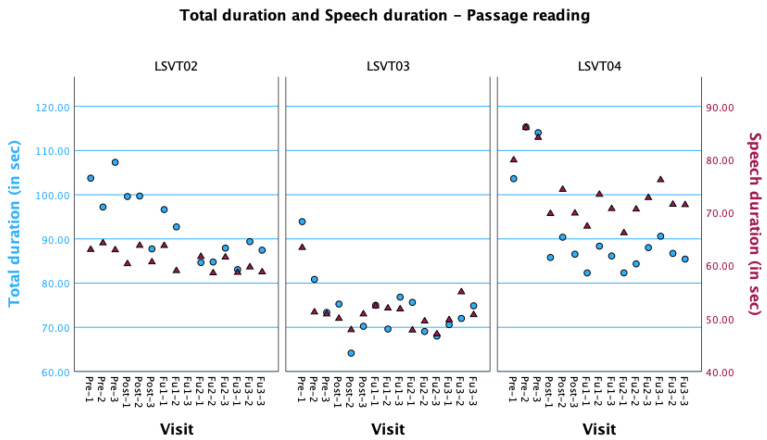
Total and speech durations values during passage reading for all participants at each visit. Blue circles, Total duration; Red triangles, Speech duration.

DDK rate was considered a control measure, i.e., was not expected to change as a result of the treatment, and remained constant over the course of this study for all participants, supporting the absence of non-specific effects of treatment. Two prosody-related measures—mean duration of speech segments and percentage of pause—improved significantly as a result of the treatment for LSVT03 and were maintained over time. These changes could result from better phonatory-respiratory coordination due to the treatment. Visual inspection of data from LSVT01 reveals a trend in total duration, whereas only an equivocal trend is present for speech duration (see Figure 5). This suggests a decrease in pausing time without an increase in articulatory rate, which could also reflect a better phonatory-respiratory coordination. It could also be due to reduced pausing as an effect of the participant becoming more familiar with the passage over time. Phonatory-respiratory incoordination has been reported in patients with PPAOS [10,55,56]. Difficulties with phonatory-respiratory coordination (getting out of breath while speaking) was an important complaint for LSVT03 and was also reported to a lesser extent for LSVT01.

These results partially align with previous results in both LSVT LOUD^®^ and PAOS literature. As in previous studies using LSVT LOUD^®^ as a treatment, there was an improvement in voice quality and control of phonation on various trained tasks for the participants, with sometimes limited generalization or diminution of the gains over time. Moreover, gains in overall confidence during speech have been reported before [59]. Regarding the literature on motor speech treatment in PAOS, none had investigated voice quality before. Direct comparison to these studies is also difficult because of the lack of overlap in measures. Combined with our results, they suggest that aspects of repeated practice could improve communication for those who suffer from progressive AOS [12]. No previous treatment study has targeted the abilities we addressed in the present study. Given the gains seen in articulation seen in previous studies on LSVT in other neurological diseases, it would be interesting to see if the same kind of result can be seen in AOS. Given the Disrupted functional connectivity seen in speech and proprioceptive circuits observed in PPAOS [60], it is possible that the overall activation of the motor system observed following LSVT treatment might have similar effects. Measures of articulation, such as vowel space, spectral moments and MFCC or kinematic measures, could capture the changes in articulation that might result from LSVT treatment in speakers with PPAOS. Studies focusing on the precise articulatory deficits in PPAOS are needed to better identify these measures. Moreover, these studies would help identify the specific factors that are related to decreased intelligibility in patients with PPAOS. Despite the limitations of the previous studies, their positive results suggest that behavioral interventions could be used to treat articulatory deficits caused by apraxia of speech in a neurodegenerative context. In addition, reading aloud, repetitive practice with words and phrases and treatment focusing on lexical and phrasal stress may improve the naturalness of speech for those with PPAOS [61]. Future studies should focus on quantitative measures of articulation (such as acoustic or kinematic measures) to confirm the improvement in articulation in patients with PAOS following speech therapy.

In addition to the abovementioned limitations, some limitations of the present study and the steps undertaken to minimize their effects are noteworthy. First, the study design presents some inherent limitations. Using a case study design cannot control for all external factors such as events in the life of the patient and historical events. To mitigate these possible effects, all participants followed the same protocol, with the same clinician and the same duration of baseline phase, intervention phase, and follow-up phase. Three baseline visits were completed for all participants, and three sessions were completed at all timepoints. For outcomes that are measured as a single value per visit (e.g., total duration during passage reading), this can limit the statistical power of the Tau-U statistics. Whenever possible, multiple values were used, i.e., tasks with multiple trials such as pitch glides, values from all individual trials were used.

Second, patient LSVT03 was evaluated remotely using Zoom videoconference software (version 5.13.0). To mitigate the potential alterations of the speech signal, the patient was sent an external microphone and was asked to use the same setup every time. Zoom settings were set to minimize audio enhancements and noise reduction algorithms (“Suppress background noise” was set to “Low,” “High fidelity music mode” was enabled, and the “Original sound” feature was used). The limitations of using a remote assessment method were also mitigated by the fact that the participant was compared to himself over time and was always recorded remotely with the same setup and equipment.

The limitations of the design also require caution in the interpretation of the results, as it is not possible to exclude all other alternative explanations to improvements in our patients (natural variation, practice effects), although steps were taken to reduce their likelihood. Likewise, it is not possible to determine with certainty the origin of the symptoms in our patients. Despite previous reports of impairments in voice modulations in patients with AOS, and the possibility that the decrease in voice quality is secondary to a suboptimal use of laryngeal function or compensation mechanism, the possible concomitance of dysarthria in our patients makes it difficult to determine if the treatment if improving features directly related to AOS or to a possible dysarthria.

Finally, it is possible that the lack of improvement on some measures such as the coefficient of variation in intensity and pitch is related to a performance that was still within normal limits in some patients. Given the lack of normative data in Quebec French relative to the expected measures, it is not possible to determine for certain that these abilities were objectively affected.

## 5. Conclusions

The present study is a first step towards investigating possible strategies to improve phonatory control in speakers with PPAOS. Given the nature of this study and the heterogeneity of profiles in patients with PPAOS, it is not possible to generalize our results to all patients with PPAOS. Nonetheless, our results of the preliminary study suggest that LSVT LOUD^®^ could be a promising tool to address certain features of phonatory control in some speakers with PPAOS, with gains that could last over two months after the end of the treatment for some aspects. Improvements in voice intensity, voice quality, phonatory control, and phono-respiratory coordination were noted. Despite all patients reporting improvements in their daily lives, generalization of the gains to more ecological tasks during the assessment were variable across participants, with an effect of the complexity of the task. The participants were satisfied with the treatment and reported increased confidence and intelligibility. The functional impact of the treatment remains to be quantitatively evaluated.

## Figures and Tables

**Table 1:** Measures of treatment outcomes on different speech systems.

Speech System	Ability	Measures	Tasks (Primary Outcomes)	Tasks (Secondary Outcomes)
Phonation	Control of loudness	Mean intensity (dB SPL)	Sustained phonation; Personalized sentences; Conversation	Standard sentences; Picture description
	Control of pitch	Maximum and maximum F0 (semitones re 1 Hz);	Pitch glides upwards;	
		F0 range (semitones)	Pitch glide downwards	
	Voice quality	AVQI		Sustained phonation and passage reading
		CPPS;	Sustained phonation	
		Jitter (%);		
		Shimmer (%);		
		HNR		
Articulation	Maximum articulation speed	DDK rates (syl/sec)		AMR—/pa/, /ta/, /ka/; SMR—/pataka/
Prosody	Variations in pitch	Coefficient of variation of pitch (semitones);		Picture description;
	Variations in loudness	Coefficient of variation of intensity (dB SPL)		Conversation;
				Standardized sentences;
				Personalized sentences
Prosody-respiration	Speech timing	Speaking rate (syl/sec);		Passage reading
		Length of articulatory groups;		
		Mean speech duration (sec);		
		Percentage of pause		

Note. AMR, alternative motion rate; AVQI, acoustic voice quality index; dB SLP, decibel of sound pressure level; CPPS, smoothed cepstral peak prominence; HNR, harmonic-to-noise ratio; sec, seconds; SMR; sequential motion rate; syl, syllables.

**Table 2:** Results for primary and secondary outcomes for patient LSVT01.

	Measures	Tasks	Post-Tx	FU1	FU2	FU3
Primary	Mean intensity (dB SPL)	Sustained phonation	✔	✔	✔	✔
outcomes		Personalized sentences	✔	✔	✔	✔
		Conversation	✔	✔	✔	
	Minimum F0 (ST re 1 Hz)	Pitch glides	✔	✔	✔	✔
	Maximum F0 (ST re 1 Hz)					
	F0 range (ST)		✔	✔	✔	✔
	CPPS	Sustained phonation	✔	✔	✔	✔
	Jitter (%)		✔			
	Shimmer (%)		✔			
	HNR		✔			
Secondary	Mean intensity (dB SPL)	Standardized sentences	✔	✔	✔	✔
outcomes		Picture description	✔	✔	✔	
	AVQI	Sustained phonation and passage reading	✔	✔	✔	
	CV of F0 (ST)	Personalized sentences				
		Standardized sentences	✔	✔	✔	✔
		Conversation				
		Picture description				
	CV of intensity (dB SPL)	Personalized sentences	✔	✔		
		Standardized sentences				
		Conversation	✔	✔	✔	
		Picture description	✔	✔		
	Speaking rate (syl/s)	Passage reading				
	Length of articulatory groups					
	Mean speech duration (s)					
	Percentage of pause					
	DDK rates (syl/s)	AMR—/pa/, /ta/, /ka/				
		SMR—/pataka/				

Note. Post-Tx, post-treatment; FU1, follow-up 1–1 week; FU2, follow-up 2–4 weeks; FU3, follow-up 3–8 weeks; dB, decibels; F0, fundamental frequency; ST, semitones; CPPS, smoothed cepstral peak prominence; CV, coefficient of variation; syl, syllable; sec, second; DDK, diadococinesias. Check marks indicate significant change. No checkmark indicates no significant change.

**Table 3:** Results for primary and secondary outcomes for patient LSVT02.

	Measures	Tasks	Post-Tx	FU1	FU2	FU3
Primary	Mean intensity (dB SPL)	Sustained phonation	✔	✔	✔	✔
outcomes		Personalized sentences	n.a.	n.a.	n.a.	n.a.
		Conversation				
	Minimum F0 (ST re 1 Hz)	Pitch glides				
	Maximum F0 (ST re 1 Hz)					
	F0 range (ST)		✔	✔	✔	✔
	CPPS	Sustained phonation	✔	✔	✔	✔
	Jitter (%)		✔	✔	✔	✔
	Shimmer (%)		✔	✔	✔	✔
	HNR		✔	✔	✔	✔
Secondary	Mean intensity (dB SPL)	Standardized sentences	✔	✔	✔	✔
outcomes		Picture description				
	AVQI	Sustained phonation and passage reading	✔	✔	✔	✔
	CV of F0 (ST)	Personalized sentences				
		Standardized sentences				
		Conversation				
		Picture description				
	CV of intensity (dB SPL)	Personalized sentences				
		Standardized sentences				
		Conversation				
		Picture description				
	Speaking rate (syl/sec)	Passage reading				
	Length of articulatory groups					
	Mean speech duration (sec)					
	Percentage of pause					
	DDK rates (syl/sec)	AMR—/pa/, /ta/, /ka/				
		SMR—/pataka/				

Note. Post-Tx, post-treatment; FU1, follow-up 1–1 week; FU2, follow-up 2–4 weeks; FU3, follow-up 3–8 weeks; dB, decibels; F0, fundamental frequency; ST, semitones; CPPS, smoothed cepstral peak prominence; CV, coefficient of variation; syl, syllable; sec, second; DDK, diadococinesias; n.a., not available. Check marks indicate significant change. No checkmark indicates no significant change.

**Table 4:** Results for primary and secondary outcomes for patient LSVT03.

	Measures	Tasks	Post-Tx	FU1	FU2	FU3
Primary	Mean intensity (dB SPL)	Sustained phonation	✔	✔	✔	✔
outcomes		Personalized sentences	✔			
		Conversation				
	Minimum F0 (ST re 1 Hz)	Pitch glides	✔	✔	✔	✔
	Maximum F0 (ST re 1 Hz)		✔	✔	✔	✔
	F0 range (ST)		✔	✔	✔	✔
	CPPS	Sustained phonation				
	Jitter (%)		✔	✔	✔	
	Shimmer (%)		✔	✔	✔	✔
	HNR		✔		✔	✔
Secondary	Mean intensity (dB SPL)	Standardized sentences				
outcomes		Picture description				
	AVQI	Sustained phonation and passage reading	✔	✔	✔	✔
	CV of F0 (ST)	Personalized sentences				
		Standardized sentences	✔			
		Conversation				
		Picture description				
	CV of intensity (dB SPL)	Personalized sentences	✔	✔	✔	
		Standardized sentences				
		Conversation				
		Picture description				
	Speaking rate (syl/sec)	Passage reading				
	Length of articulatory groups					
	Mean speech duration (sec)		✔	✔	✔	✔
	Percentage of pause		✔	✔	✔	✔
	DDK rates (syl/sec)	AMR—/pa/, /ta/, /ka/				
		SMR—/pataka/				

Note. Post-Tx, post-treatment; FU1, follow-up 1–1 week; FU2, follow-up 2–4 weeks; FU3, follow-up 3–8 weeks; dB, decibels; F0, fundamental frequency; ST, semitones; CPPS, smoothed cepstral peak prominence; CV, coefficient of variation; syl, syllable; sec, second; DDK, diadococinesias. Check marks indicate significant change. No checkmark indicates no significant change.

## Data Availability

Quantitative data from acoustic analyses can be provided upon reasonable request.

## Supplementary Materials

The following supporting information can be downloaded at: https://www.mdpi.com/article/10.3390/brainsci14050417/s1. Table S1. Tau-U results for mean intensity. Table S2. Tau-U results for pitch control during pitch glides. Table S3. Tau-U statistics for voice quality measures. Table S4. Prosody—speech and pause measures. Table S5. Prosody—modulations of intensity and pitch during connected speech.
