# Supplementary material for: Efficacy of LSVT LOUD® on Phonatory Control and Voice Quality in Patients with Primary Progressive Apraxia of Speech: Case Studies
Source: Brain Sci. 2024 Apr 24;14(5):417. doi: 10.3390/brainsci14050417 (PMC11117832; doi:10.3390/brainsci14050417)
Supplement: Supplementary file 1 [file brainsci-14-00417-s001.zip › brainsci-2898336-supplementary.pdf]

Supplementary Materials

Table S1. Tau-U results for Mean Intensity

Participant	Phase	Sustained vowel	Standardized sentences	Personalized sentences	Conversation	Picture description
LSVT01	Pre-Post	**Tau-U = 0.50, *p* = 0.009**	**Tau-U = 0.40, *p* < 0.001**	**Tau-U = 0.22, *p* = 0.014**	**Tau-U = 1, *p* = 0.030**	**Tau-U = 1, *p* = 0.032**
	Pre-FU1	**Tau-U = 0.50, *p* = 0.003**	**Tau-U = 0.48, *p* < 0.001**	**Tau-U = 0.33, *p* < 0.001**	**Tau-U = 1, *p* = 0.018**	**Tau-U = 1, *p* = 0.015**
	Pre-FU2	**Tau-U = 0.50, *p* = 0.006**	**Tau-U = 0.31, *p* < 0.001**	**Tau-U = 0.21, *p* = 0.019**	**Tau-U = 1, *p* = 0.018**	**Tau-U = 1, *p* = 0.018**
	Pre-FU3	**Tau-U = 0.50, *p* = 0.029**	**Tau-U = 0.42, *p* < 0.001**	**Tau-U = 0.41, *p* < 0.001**	Tau-U = 0.83, *p* = 0.059	Tau-U = 0.83, *p* = 0.063
LSVT02	Pre-Post	**Tau-U = 0.44, *p* = 0.012**	**Tau-U = 0.32, *p* = 0.001**	n.a.	Tau-U = -0.17, *p* = 0.695	Tau-U = 0.56, *p* = 0.157
	Pre-FU1	**Tau-U = 0.44, *p* = 0.008**	**Tau-U = 0.21, *p* = 0.031**	n.a.	Tau-U = 0.17, *p* = 0.764	Tau-U = 0.44, *p* = 0.278
	Pre-FU2	**Tau-U = 0.44, *p* = 0.010**	**Tau-U = 0.21, *p* = 0.034**	n.a.	Tau-U = 0.5, *p* = 0.224	Tau-U = 0.78, *p* = 0.059
	Pre-FU3	**Tau-U = 0.37, *p* = 0.033**	**Tau-U = -0.37, *p* < 0.001**	n.a.	Tau-U = 0.06, *p* = 0.970	Tau-U = 0.56, *p* = 0.156
LSVT03	Pre-Post	**Tau-U = 0.56, *p* = 0.001**	Tau-U = 0.12, *p* = 0.206	**Tau-U = 0.34, *p* < 0.001**	Tau-U = 0.50, *p* = 0.320	Tau-U = 0.50, *p* = 0.224
	Pre-FU1	**Tau-U = 0.56, *p* = 0.001**	Tau-U = -0.09, *p* = 0.331	**Tau-U = 0.24, *p* = 0.007**	Tau-U = 0.50, *p* = 0.220	Tau-U = 0.50, *p* = 0.219
	Pre-FU2	**Tau-U = 0.56, *p* = 0.001**	**Tau-U = -0.22, *p* = 0.016**	Tau-U = 0.14, *p* = 0.127	Tau-U = 0.50, *p* = 0.316	Tau-U = 0.50, *p* = 0.221
	Pre-FU3	**Tau-U = 0.56, *p* = 0.001**	Tau-U = 0.17, *p* = 0.066	**Tau-U = 0.32, *p* < 0.001**	Tau-U = 0.50, *p* = 0.223	Tau-U = 0.50, *p* = 0.221

Note. CoV, coefficient of variation; dB, decibels; ST, semitones; Pre, Pre-treatment; Post, Post-treatment; FU1, follow-up 1 (1 week); FU2, follow-up 2 (4 weeks); FU3, follow-up 3, (8 weeks); n.a., not available. Significant results (p>0.05) shown in bold.

Table S2. Tau-U results for Pitch Control during Pitch Glides

Participant	Phases	Max F0	Range upwards	Min F0	Range downwards
LSVT01	Pre-Post	Tau-U = 0.116, p = 0.536	**Tau-U = -0.401, p = 0.027**	**Tau-U = -0.657, p < 0.000**	Tau-U = -0.234, p = 0.075
	Pre-FU1	Tau-U = 0.286, p = 0.112	Tau-U = -0.3, p = 0.090	**Tau-U = -0.713, p < 0.000**	**Tau-U = -0.27, p = 0.026**
	Pre-FU2	Tau-U = 0.301, p = 0.100	Tau-U = -0.352, p = 0.051	**Tau-U = -0.75, p < 0.000**	**Tau-U = -0.29, p = 0.021**
	Pre-Fu3	Tau-U = 0.343, p = 0.070	Tau-U = -0.357, p = 0.052	**Tau-U = -0.75, p < 0.000**	**Tau-U = -0.461, p = 0.003**
LSVT02	Pre-Post	Tau-U = 0.161, p = 0.320	Tau-U = -0.036, p = 0.841	Tau-U = 0.278, p = 0.140	**Tau-U = -0.544, p = 0.003**
	Pre-FU1	Tau-U = 0.177, p = 0.284	Tau-U = 0.034, p = 0.858	Tau-U = -0.142, p = 0.445	**Tau-U = -0.355, p = 0.048**
	Pre-FU2	**Tau-U = 0.355, p = 0.030**	Tau-U = 0.164, p = 0.335	Tau-U = 0.13, p = 0.486	**Tau-U = -0.515, p = 0.003**
	Pre-Fu3	Tau-U = 0.194, p = 0.240	Tau-U = -0.036, p = 0.838	**Tau-U = 0.5, p = 0.005**	**Tau-U = 0.357, p = 0.003**
LSVT03	Pre-Post	Tau-U = 0.154, p = 0.409	**Tau-U = 0.401, p = 0.028**	**Tau-U = 0.625, p = 0.001**	Tau-U = 0.005, p = 0.999
	Pre-FU1	**Tau-U = 0.500, p = 0.006**	**Tau-U = 0.463, p = 0.009**	**Tau-U = 0.625, p < 0.000**	Tau-U = -0.02, p = 0.912
	Pre-FU2	Tau-U = 0.333, p = 0.070	**Tau-U = 0.486, p = 0.008**	**Tau-U = 0.625, p < 0.000**	**Tau-U = 0.375, p = 0.036**
	Pre-Fu3	**Tau-U = 0.500, p = 0.005**	**Tau-U = 0.488, p = 0.008**	**Tau-U = 0.625, p < 0.000**	**Tau-U = 0.526, p < 0.000**

Note. Max F0, maximum fundamental frequency; Min F0, minimum fundamental frequency; Pre, Pre-treatment; Post, Post-treatment; FU1, follow-up 1 (1 week); FU2, follow-up 2 (4 weeks); FU3, follow-up 3, (8 weeks). Statistically significant results (p > 0.05) shown in bold.

Table S3. Tau-*U* statistics for voice quality measures.

Participant	Phase	AVQI	CPPS	HNR	Shimmer%	Shimmer dB	Jitter	Median pitch
LSVT01	Pre-Post	**Tau-U = -0.50, $p = 0.010$**	**Tau-U = 0.50, $p = 0.005$**	**Tau-U = 0.50, $p = 0.005$**	**Tau-U = -0.49, $p = 0.006$**	**Tau-U = -0.50, $p = 0.006$**	**Tau-U = -0.46, $p = 0.009$**	**Tau-U = 0.56, $p = 0.001$**
	Pre-FU1	**Tau-U = -0.50, $p = 0.004$**	**Tau-U = 0.50, $p = 0.006$**	**Tau-U = 0.50, $p = 0.005$**	**Tau-U = -0.50, $p = 0.005$**	**Tau-U = -0.50, $p = 0.005$**	**Tau-U = -0.46, $p = 0.0092$**	**Tau-U = 0.60, $p < 0.001$**
	Pre-FU2	**Tau-U = -0.50, $p = 0.030$**	**Tau-U = 0.50, $p = 0.004$**	**Tau-U = 0.50, $p = 0.006$**	**Tau-U = -0.41, $p = 0.0211$**	**Tau-U = -0.40, $p = 0.025$**	**Tau-U = -0.45, $p = 0.0133$**	**Tau-U = 0.61, $p < 0.001$**
	Pre-FU3	Tau-U = -0.17, $p = 0.706$	**Tau-U = 0.50, $p = 0.029$**	Tau-U 0.17, $p = 0.502$	Tau-U = -0.17, $p = 0.481$	Tau-U = -0.17, $p = 0.490$	Tau-U = -0.07, $p = 0.716$	**Tau-U = 0.50, $p = 0.009$**
LSVT02	Pre-Post	**Tau-U = -0.75, $p < 0.001$**	**Tau-U = 0.63, $p < 0.002$**	**Tau-U = 0.61, $p < 0.001$**	**Tau-U = -0.61, $p < 0.001$**	**Tau-U = -0.61, $p < 0.001$**	**Tau-U = -0.50, $p = 0.007$**	**Tau-U = 0.63, $p < 0.001$**
	Pre-FU1	**Tau-U = -0.75, $p < 0.001$**	**Tau-U = 0.63, $p < 0.001$**	**Tau-U = 0.60, $p < 0.001$**	**Tau-U = -0.61, $p < 0.001$**	**Tau-U = -0.61, $p < 0.001$**	**Tau-U = -0.50, $p = 0.005$**	**Tau-U = 0.46, $p = 0.009$**
	Pre-FU2	**Tau-U = -0.75, $p < 0.001$**	**Tau-U = 0.64, $p < 0.001$**	**Tau-U = 0.58, $p = 0.001$**	**Tau-U = -0.50, $p = 0.005$**	**Tau-U = -0.49, $p < 0.007$**	**Tau-U = -0.50, $p = 0.006$**	**Tau-U = 0.53, $p = 0.003$**
	Pre-FU3	**Tau-U = -0.75, $p < 0.001$**	**Tau-U = 0.63, $p < 0.001$**	**Tau-U = 0.600, $p < 0.001$**	**Tau-U = -0.61, $p < 0.001$**	**Tau-U = -0.61, $p < 0.001$**	**Tau-U = -0.50, $p = 0.005$**	**Tau-U = 0.625, $p < 0.001$**
LSVT03	Pre-Post	**Tau-U = --0.39, $p = 0.025$**	Tau-U = 0.24, $p = 0.193$	**Tau-U = 0.36, $p = 0.043$**	**Tau-U = -0.45, $p = 0.013$**	**Tau-U = -0.45, $p = 0.013$**	**Tau-U = -0.50, $p = 0.005$**	Tau-U = 0.24, $p = 0.185$
	Pre-FU1	**Tau-U = --0.39, $p = 0.025$**	Tau-U = 0.23, $p = 0.215$	Tau-U = 0.338, $p = 0.067$	**Tau-U = -0.40 $p = 0.027$**	**Tau-U = -0.44, $p = 0.014$**	**Tau-U = -0.50, $p = 0.005$**	Tau-U = 0.30, $p = 0.098$
	Pre-FU2	**Tau-U = --0.37, $p = 0.030$**	**Tau-U = 0.38, $p = 0.034$**	**Tau-U = -0.50, $p = 0.007$**	**Tau-U = -0.50, $p = 0.007$**	Tau-U = 0.13, $p = 0.501$	**Tau-U = 0.48, $p = 0.008$**	**Tau-U = -0.50, $p = 0.006$**
	Pre-FU3	**Tau-U = -0.36, $p = 0.031$**	**Tau-U = 0.36, $p = 0.044$**	**Tau-U = -0.40, $p = 0.029$**	**Tau-U = -0.48, $p = 0.007$**	Tau-U = 0.04, $p = 0.832$	Tau-U = 0.28, $p = 0.132$	**Tau-U = -0.50, $p = 0.005$**

Note. AVQI, Acoustic Voice Quality Index; CPPS, Cepstral Peak Prominence Smoothed; HNR, Harmonic to Noise Ratio; dB, decibels; Pre, Pre-treatment; Post, Post-treatment; FU1, follow-up 1 (1 week); FU2, follow-up 2 (4 weeks); FU3, follow-up 3, (8 weeks). Statistically significant results ($p > 0.05$) shown in bold

Table S4. Prosody – Speech and Pause measures

Participant	Phase	Percentage of pause	Speech duration	Total duration	Mean pause duration	Mean speech duration
LSVT01	Pre-post	Tau-U = -0.333, $p = 0.459$	Tau-U = -0.166, $p = 0.692$	Tau-U = -0.333, $p = 0.465$	Tau-U = -0.666, $p = 0.081$	Tau-U = 0.166, $p = 0.831$
	Pre-FU1	Tau-U = -0.277, $p = 0.536$	Tau-U = -0.277, $p = 0.535$	Tau-U = -0.500, $p = 0.226$	Tau-U = -0.666, $p = 0.076$	Tau-U = 0.166, $p = 0.763$
	Pre-FU2	Tau-U = -0.500, $p = 0.227$	Tau-U = -0.500, $p = 0.216$	Tau-U = -0.500, $p = 0.227$	**Tau-U = -0.888, $p = 0.030$**	Tau-U = 0.277, $p = 0.531$
	Pre-FU3	Tau-U = -0.500, $p = 0.319$	Tau-U = -0.500, $p = 0.315$	Tau-U = -0.500, $p = 0.325$	Tau-U = -0.833, $p = 0.061$	Tau-U = 0.166, $p = 0.836$
LSVT02	Pre-post	Tau-U = -0.277, $p = 0.542$	Tau-U = 0.111, $p = 0.781$	Tau-U = 0.111, $p = 0.781$	Tau-U = 0.055, $p = 0.970$	Tau-U = 0.500, $p = 0.225$
	Pre-FU1	Tau-U = -0.277, $p = 0.543$	Tau-U = 0.666, $p = 0.114$	Tau-U = 0.222, $p = 0.579$	Tau-U = 0.388, $p = 0.348$	Tau-U = 0.500, $p = 0.220$
	Pre-FU2	Tau-U = -0.055, $p = 0.968$	**Tau-U = 0.000, $p = 0.028$**	Tau-U = 0.111, $p = 0.786$	Tau-U = -0.166, $p = 0.684$	Tau-U = 0.388, $p = 0.343$
	Pre-FU3	Tau-U = -0.388, $p = 0.322$	Tau-U = 0.222, $p = 0.573$	Tau-U = 0.111, $p = 0.780$	Tau-U = 0.500, $p = 0.210$	Tau-U = 0.500, $p = 0.217$
LSVT03	Pre-post	**Tau-U = -1.00, $p = 0.018$**	Tau-U = -0.500, $p = 0.224$	Tau-U = -0.500, $p = 0.218$	Tau-U = -0.500, $p = 0.212$	**Tau-U = 1.00, $p = 0.020$**
	Pre-FU1	**Tau-U = -1.00, $p = 0.016$**	Tau-U = -0.500, $p = 0.227$	Tau-U = -0.500, $p = 0.213$	Tau-U = -0.500, $p = 0.210$	**Tau-U = 1.00, $p = 0.014$**
	Pre-FU2	**Tau-U = -1.00, $p = 0.015$**	Tau-U = -0.500, $p = 0.220$	Tau-U = -0.500, $p = 0.222$	Tau-U = -0.166, $p = 0.687$	**Tau-U = 1.00, $p = 0.017$**
	Pre-FU3	**Tau-U = -1.00, $p = 0.016$**	Tau-U = -0.500, $p = 0.224$	Tau-U = -0.500, $p = 0.227$	Tau-U = -0.500, $p = 0.233$	**Tau-U = 1.00, $p = 0.016$**

Note. Pre, Pre-treatment; Post, Post-treatment; FU1, follow-up 1 (1 week); FU2, follow-up 2 (4 weeks); FU3, follow-up 3, (8 weeks). Statistically significant results ($p > 0.05$) shown in bold

Table S5. Prosody – modulations of intensity and pitch during connected speech

Participant	Task	Phase	CoV Intensity (dB)	CoV pitch (ST)
LSVT01	Standardized sentences	Pre-Post	**Tau-U = -0.20, *p* = 0.032**	**Tau-U = -0.36, *p* < 0.001**
		Pre-FU1	**Tau-U = -0.24, *p* = 0.008**	**Tau-U = -0.27, *p* = 0.004**
		Pre-FU2	Tau-U = -0.16, *p* = 0.083	**Tau-U = -0.33, *p* < 0.001**
		Pre-FU3	Tau-U = -0.03, *p* = 0.760	**Tau-U = -0.33, *p* = 0.001**
	Personalized sentences	Pre-Post	Tau-U = -0.01, *p* = 0.884	Tau-U = 0.03, *p* = 0.725
		Pre-FU1	Tau-U = -0.16, *p* = 0.083	Tau-U = 0.05, *p* = 0.593
		Pre-FU2	Tau-U = 0.06, *p* = 0.495	Tau-U = -0.05, *p* = 0.583
		Pre-FU3	Tau-U = 0.15, *p* = 0.133	Tau-U = -0.08, *p* = 0.448
	Conversation	Pre-Post	Tau-U = -0.17, *p* = 0.688	Tau-U = 0.17, *p* = 0.827
		Pre-FU1	Tau-U = -0.17, *p* = 0.681	Tau-U = 0.06, *p* = 0.967
		Pre-FU2	Tau-U = 0.06, *p* = 0.971	Tau-U = 0.39, *p* = 0.347
		Pre-FU3	Tau-U = 0.33, *p* = 0.469	Tau-U = -0.17, *p* = 0.693
	Picture description	Pre-Post	**Tau-U = 1.00, *p* = 0.032**	Tau-U = 0.17, *p* = 0.829
		Pre-FU1	**Tau-U = 0.89, *p* = 0.032**	Tau-U = 0.06, *p* = 0.973
		Pre-FU2	**Tau-U = 0.89, *p* = 0.032**	Tau-U = 0.39, *p* = 0.336
		Pre-FU3	Tau-U = 0.00, *p* = 1.000	Tau-U = -0.17, *p* = 0.688
LSVT02	Standardized sentences	Pre-Post	Tau-U = -0.06, *p* = 0.513	Tau-U = 0.07, *p* = 0.502
		Pre-FU1	Tau-U = -0.17, *p* = 0.089	Tau-U = 0.12, *p* = 0.216
		Pre-FU2	Tau-U = 0.07, *p* = 0.512	Tau-U = 0.04, *p* = 0.682
		Pre-FU3	Tau-U = 0.03, *p* = 0.748	**Tau-U = 0.20, *p* = 0.035**
	Personalized sentences	Pre-Post	n.a.	n.a.
		Pre-FU1	n.a.	n.a.
		Pre-FU2	n.a.	n.a.
		Pre-FU3	n.a.	n.a.
	Conversation	Pre-Post	Tau-U = -0.17, *p* = 0.695	Tau-U = -0.5, *p* = 0.323
		Pre-FU1	Tau-U = -0.06, *p* = 0.968	Tau-U = -0.17, *p* = 0.683
		Pre-FU2	Tau-U = 0.17, *p* = 0.759	Tau-U = -0.17, *p* = 0.687
		Pre-FU3	Tau-U = -0.06, *p* = 0.971	Tau-U = 0.06, *p* = 0.969
	Picture description	Pre-Post	Tau-U = 0.17, *p* = 0.759	Tau-U = 0.17, *p* = 0.760
		Pre-FU1	Tau-U = 0.17, *p* = 0.763	Tau-U = 0.17, *p* = 0.761
		Pre-FU2	Tau-U = 0.17, *p* = 0.763	Tau-U = 0.28, *p* = 0.537
		Pre-FU3	Tau-U = 0.17, *p* = 0.757	Tau-U = 0.28, *p* = 0.536
LSVT03	Standardized sentences	Pre-Post	Tau-U = 0.16, *p* = 0.087	**Tau-U = 0.21, *p* = 0.026**
		Pre-FU1	Tau-U = 0.09, *p* = 0.343	Tau-U = 0.01, *p* = 0.961

	Pre-FU2	Tau-U = 0.14, p = 0.142	Tau-U = -0.08, p = 0.399
	Pre-FU3	Tau-U = 0.08, p = 0.370	Tau-U = 0.14, p = 0.129
Personalized sentences	Pre-Post	**Tau-U = -0.21, p = 0.009**	Tau-U = 0.08, p = 0.368
	Pre-FU1	**Tau-U = -0.30, p < 0.001**	Tau-U = -0.002, p = 0.980
	Pre-FU2	**Tau-U = -0.26, p = 0.001**	Tau-U = -0.04, p = 0.622
	Pre-FU3	Tau-U = -0.14, p = 0.077	Tau-U = 0.08, p = 0.345
Conversation	Pre-Post	Tau-U = 0.50, p = 0.316	Tau-U = 0.17, p = 0.723
	Pre-FU1	Tau-U = 0.50, p = 0.218	Tau-U = 0.56, p = 0.143
	Pre-FU2	Tau-U = 0.50, p = 0.322	Tau-U = 0.00, p = 1.000
	Pre-FU3	Tau-U = 0.50, p = 0.221	Tau-U = 0.22, p = 0.572
Picture description	Pre-Post	Tau-U = 0.28, p = 0.546	Tau-U = 0.39, p = 0.353
	Pre-FU1	Tau-U = 0.28, p = 0.535	Tau-U = 0.06, p = 0.969
	Pre-FU2	Tau-U = -0.17, p = 0.685	Tau-U = -0.17, p = 0.687
	Pre-FU3	Tau-U = 0.39, p = 0.343	Tau-U = 0.50, p = 0.219

Note. CoV, coefficient of variation; dB, decibels; ST, semitones; Pre, Pre-treatment; Post, Post-treatment; FU1, follow-up 1 (1 week); FU2, follow-up 2 (4 weeks); FU3, follow-up 3, (8 weeks); n.a., not available. Statistically significant results (p > 0.05) shown in bold.
